# Telemonitoring and Case Management for Hypertensive and Remote-Dwelling Patients With Chronic Kidney Disease—The Telemonitoring for Improved Kidney Outcomes Study (TIKO): A Clinical Research Protocol

**DOI:** 10.1177/20543581221077500

**Published:** 2022-02-13

**Authors:** Ikechi G. Okpechi, Deenaz Zaidi, Feng Ye, Miriam Fradette, Kara Schick-Makaroff, Charlotte Berendonk, Abdullah Abdulrahman, Branko Braam, Anukul Ghimire, Vinash Kumar Hariramani, Kailash Jindal, Maryam Khan, Scott Klarenbach, Shezel Muneer, Jennifer Ringrose, Nairne Scott-Douglas, Soroush Shojai, Dan Slabu, Naima Sultana, Mohammed M. Tinwala, Stephanie Thompson, Raj Padwal, Aminu K. Bello

**Affiliations:** 1 Division of Nephrology and Immunology, Department of Medicine, University of Alberta, Edmonton, Canada; 2 University of Alberta, Edmonton, Canada

**Keywords:** blood pressure, cardiovascular disease, chronic kidney disease, hypertension, remote dwelling, telemonitoring

## Abstract

**Background:**

Hypertension, together with poorly controlled blood pressure (BP) are known risk factors for kidney disease and progression to kidney failure as well as increased cardiovascular (CV) morbidity and mortality. Several studies in patients without kidney disease have demonstrated the efficacy of home BP telemonitoring (HBPT) for BP control.

**Objective:**

The primary aim of this study is to assess the mean difference in systolic BP (SBP) at 12 months, from baseline in remote dwelling patients with hypertension and chronic kidney disease (CKD) in Northern Alberta, Canada, comparing HBPT + usual care versus HBPT + a case manager. Other secondary objectives, including cost-effectiveness and acceptability of HBPT as well as occurrence of adverse events will also be assessed.

**Methods:**

**Design:**

This study is designed as a pragmatic randomized controlled trial (RCT) of HBPT plus clinical case management compared to HBPT with usual care.

**Setting:**

Peace River region in Northern Alberta Region, Canada.

**Patients:**

Primary care patients with CKD and hypertension.

**Measurements:**

Eligible patients will be randomized 1:1 to HBPT + BP case management versus HBPT + usual care. In the intervention arm, BP will be measured 4 times daily for 1 week, with medications titrated up or down by the study case manager until guideline targets (systolic BP [SBP]: <130 mmHg) are achieved. Once BP is controlled, (ie, to guideline-concordant targets), this 1-week protocol will be repeated every 3 months for 1 year. Patients in the control arm will also follow the same BP measurement protocol; however, there will be no interactions with the case manager; they will share their BP readings with their primary care physicians or nurse practitioners at scheduled visits.

**Limitations:**

Potential limitations of this study include the relatively short duration of follow-up, possible technological pitfalls, and need for patients to own a smartphone and have access to the internet to participate.

**Conclusions:**

As this study will focus on a high-risk population that has been characterized by a large care gap, it will generate important evidence that would allow targeted and effective population-level strategies to be implemented to improve health outcomes for high-risk hypertensive CKD patients in Canada’s remote communities.

**Trial Registration:**

www.clinicaltrials.gov (NCT number: NCT04098354)

## Abrégé

**Contexte:**

L’hypertension et la pression artérielle (PA) mal contrôlée sont des facteurs de risque reconnus pour la néphropathie et la progression vers l’insuffisance rénale, en plus de poser un risque accru de morbidité et de mortalité cardiovasculaires. Plusieurs études chez des patients sans néphropathie ont démontré l’efficacité de la télésurveillance de la PA à domicile (TSPA) pour le contrôle de la PA.

**Objectifs:**

Le principal objectif est d’évaluer la différence moyenne de pression artérielle systolique (PAS) après 12 mois par rapport à sa valeur initiale chez des patients atteints d’hypertension et d’insuffisance rénale chronique (IRC) habitant les communautés éloignées du nord de l’Alberta (Canada). Cet objectif sera atteint en comparant la TSPA + soins habituels à la TSPA + gestionnaire de cas. D’autres objectifs secondaires, notamment le rapport coût/efficacité de la TSPA, son acceptation et la survenue d’événements indésirables seront également évalués.

**Type d’étude:**

Cette étude est conçue comme un essai randomisé contrôlé (ERC) pragmatique comparant la TSPA + prise en charge clinique des cas à la TSPA + soins habituels.

**Cadre:**

Région de Peace River dans le nord de l’Alberta (Canada).

**Sujets:**

Patients atteints d’IRC et d’hypertension recevant des soins de santé primaires.

**Mesures:**

Les patients admissibles seront répartis 1:1 dans le groupe TSPA + prise en charge du cas d’hypertension ou dans le groupe témoin (TSPA + soins habituels). Dans le groupe d’intervention, la PA sera mesurée quatre fois par jour pendant une semaine, avec augmentation ou réduction de la médication par le gestionnaire de cas de l’étude jusqu’à ce que la cible de référence (PAS : <130 mmHg) soit atteinte. Une fois la PA contrôlée (c.-à-d. conforme aux cibles recommandées), ce protocole sur une semaine sera répété tous les trois mois pendant un an. Les patients du groupe témoin suivront le même protocole de mesure de la PA, mais sans interactions avec le gestionnaire de cas, ils transmettront plutôt leurs mesures de PA à leur médecin de soins primaires ou aux infirmières praticiennes lors de visites prévues.

**Limites:**

Cette étude est notamment limitée par la durée relativement courte du suivi, de possibles difficultés technologiques et la nécessité pour les participants de posséder un téléphone intelligent et d’avoir accès à l’Internet.

**Conclusion:**

Puisque cette étude se penchera sur une population à risque élevé et marquée par d’importantes lacunes en matière de soins, elle générera des données importantes qui aideront à mettre en œuvre des stratégies ciblées et efficaces au niveau de la population afin d’améliorer les évènements cliniques des patients hypertendus et atteints d’IRC à haut risque habitant les communautés éloignées au Canada.

## Introduction

Poorly controlled blood pressure (BP) is a risk factor for rapid progression of chronic kidney disease (CKD) to kidney failure and is also associated with cardiovascular (CV) morbidity and mortality.^1-4^ Poorly controlled BP is common and often associated with a higher rate of adverse clinical outcomes among remote/rural dwellers due to limited access to and lower quality of chronic disease care.^3,5^ Recent data have demonstrated that remote dwellers with CKD have less access to specialist care, receive poorer clinical care in all aspects of the care process, and exhibit worse clinical outcomes compared to their counterparts living in urban centers.^1,6-9^ Notably, among remote dwellers with CKD, a lack of BP control is the most important identified element of evidence-based care that is amenable to intervention.^1,9^

Recent guidelines by Kidney Disease Improving Global Outcomes (KDIGO) recommend that systolic blood pressure (SBP) be lowered to <120 mmHg in all patients (***level 2B—*** if tolerated, because of potential harms and lack of evidence in special groups) but to aim for <130 mmHg or even higher if a lower target is not tolerated.^10^ Guidelines from Hypertension Canada, however, still recommend ≤130 mmHg or ≤ 120 mmHg in patients with diabetes or CKD and <140 mmHg in all other patients.^11^ BP is the most important prognostic factor for CKD clinical outcomes;^12^ however, among patients with CKD (Stages 3 and 4), only a fraction meet the recommended BP targets using existing care approaches.^4,12-15^ Contemporary BP management guidelines recommend shifting from office-based BP measurement to home/ambulatory readings because the latter are more prognostically accurate: well-conducted randomized controlled trials (RCTs) provide strong evidence of the benefits of home BP monitoring compared to conventional office measures.^2,16-21^

Several studies have demonstrated the feasibility and accuracy of home BP telemonitoring (HBPT) and increased patient satisfaction compared to the usual care among those with some chronic conditions.,^15,22^ In 1 study, when compared to usual care, the adjusted mean SBP differences with HBPT was −4·7 mm Hg (–7·0 to −2·4; *P* < .0001) and when HBPT was combined with additional care (eg, counseling, education, behavioral management, etc) the mean reduction in SBP and DBP was of a larger magnitude, suggesting that HBPT can be more efficacious when proactive additional support is provided.^23^ Findings from recent reviews and meta-analyses also show the positive impact of telemonitoring on patient outcomes for those with chronic conditions such as diabetes, asthma, and heart failure.^24-27^ One systematic review and meta-analysis on the effects of HBPT on blood pressure and kidney function in CKD patients reported significant reduction of SBP (-8.8 mmHg; [95% CI: -16.2, -1.4]; *P* = .02) and DBP (-2.4 mmHg; [-3.8, -1.0]; *P* < .001), and significant improvement in estimated glomerular filtration rate (5.35 mL/min/1.73 m^2^; [2.49, 8.21]; *P* < 0.001).^28^ Canadians living in remote communities continue to experience various challenges in accessing healthcare; lack of access to healthcare services and treatments in such settings is often compounded by geographical isolation from mainstream health services. Hence, the use of HBPT technologies could prove to be advantageous for improving control of hypertension and reducing associated target organ damage in populations in remote regions. However, the clinical efficacy, safety, acceptability, and cost-effectiveness of HBPT among CKD patients living in remote/rural communities with limited access to care is unknown.

Therefore, the primary aim of this study is to assess the mean difference in SBP at 12 months, from baseline in remote communities in Northern Alberta through a pragmatic RCT, comparing HBPT + usual care versus HBPT + a case manager.

## Methods

### Study Objectives

The primary aim of this study is to assess the mean difference in SBP at 12 months, from baseline in remote communities in Northern Alberta through a pragmatic RCT, comparing HBPT + usual care versus HBPT + a case manager. Secondary aims of the study will include:

Proportion of patients who maintained home SBP within guideline target (<130 mmHg)^10,11^ from baseline to the end of the study.
Proportion of patients who remained within guideline target SBP (<130 mmHg),^10,11^ throughout the study.
The user acceptability of HBPT for BP control, using a qualitative approach.
The cost-effectiveness and cost utility of HBPT combined with a protocol-based case management approach among remote-dwellers with CKD compared to the usual care.
5.Proportion of adverse events, eg, syncope, hypotension requiring assistance or medical attention, worsening of kidney function (2x serum creatinine or 50% decline in estimated glomerular filtration rate [eGFR]), and/or electrolyte abnormalities [hyperkalemia ≥ 6.5 mmol/L], in each arm of the study.

### Study Setting

**Figure 1.**
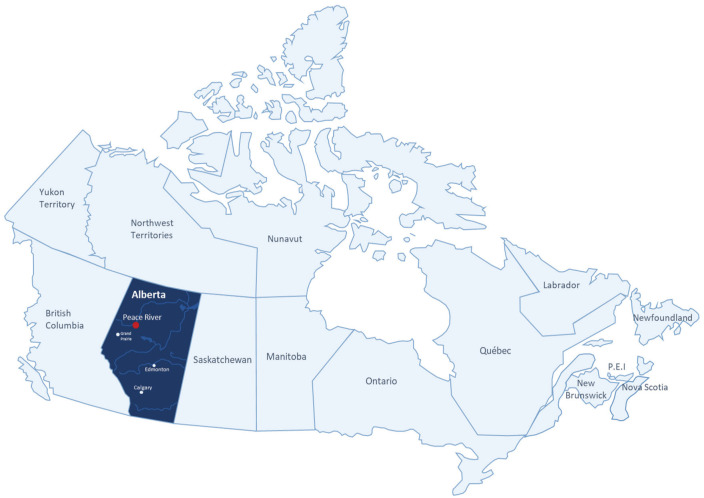
Map of Alberta showing Northern Alberta and Peace River region.

This study will be conducted in select remote communities in the Peace River Region of Northern Alberta, located ~1,000 km from Edmonton with a population base of ~75,000 (Figure 1). The CKD population in this region receives care from a primary care network consisting of 35 primary care providers (PCPs) affiliated with 9 primary care group practices. Patients with CKD and hypertension will be identified from the Northern Alberta Renal Program (NARP) database and contacted by the study team to participate in this study.

### Design, Randomization, and Allocation

**Figure 2.**
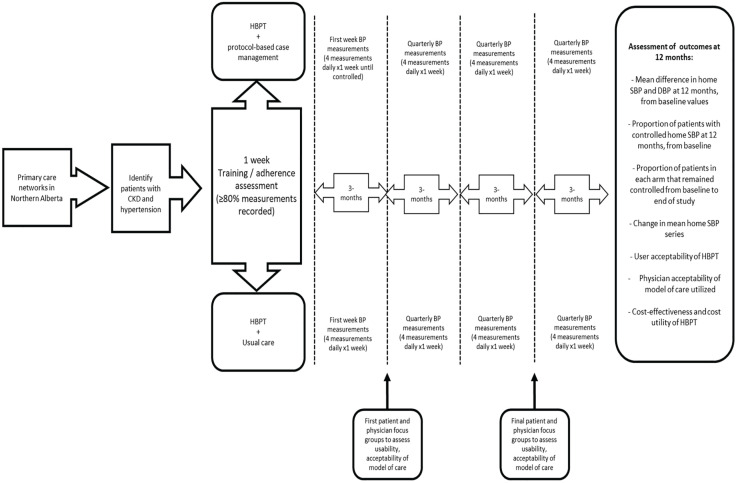
A summary of the trial design. *Note.* HBPT = home blood pressure telemonitoring; BP = blood pressure; SBP = systolic blood pressure; DBP = diastolic blood pressure; CKD = chronic kidney disease.

We have designed this study as a 2-arm pragmatic RCT comparing the clinical care of remote dwelling hypertensive CKD patients. We will randomly assign the patients with CKD and hypertension to each study arm (1:1) that is, HBPT plus protocol-based case management versus HBPT plus usual care (Figure 2). A randomized permuted block design of 4 and 6 will be used. The random allocation sequence will be computer generated using STATA 17 software (StataCorp. 2021. Stata Statistical Software: Release 17. College Station, Texas: StataCorp LLC) and allocation will be concealed by web-based central randomization using The Research Electronic Data Capture System (REDCap version 8.8.2; 2018 Vanderbilt University). Participants and clinicians will not be blinded to group assignment.

### Participant Recruitment

#### Study population

The following eligibility criteria will apply to this study:

Age ≥ 18 years with documented diagnosis of established CKD (not on dialysis with eGFR < 60 mL/min/1.73 m^2^ and/or proteinuria > 1 g/day)
Remote dwelling patients in the Peace River region of Alberta, Canada
Patients known with hypertension (physician diagnosed / self reported and currently taking antihypertensive medications)
Owning a smartphone (iOS or Android) with access to wireless internet connection.
Proficiency in English language (both verbal and written); and ability and willingness to provide informed consent for participation.
Ability and willingness to use the HBPT device (≥80% recordings sent in the training period)

The following exclusion criteria will apply to this study:

Patients with hypertensive urgency or emergency identified during the training period (immediate consultation will be initiated with the patient’s PCP or with a hypertension specialist)
Patients with stage 5 CKD (eGFR ≤ 15 ml/min/1.73 m)^2^ or patients receiving kidney replacement therapy
Heart failure with reduced ejection fraction
Presence of any terminal illness (life expectancy < 1 year)
Participation in any ongoing clinical drug trial
Pregnancy, lactation/breastfeeding
Planning to relocate out of the Peace River region or residence in an area without mobile phone coverage.

#### Intervention arm

**Figure 3.**
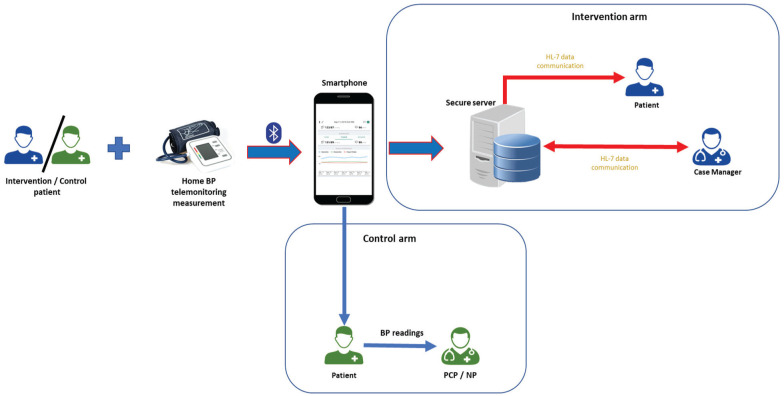
Working prototype of a home BP telemonitoring system. *Note.* BP = blood pressure Intervention / control patients measures BP at home. Information is transferred wirelessly from the BP monitor to the smartphone via a Bluetooth transmission protocol. The smartphone receives the information, organizes, and displays it on the smartphone, and encrypts the information for further transmission over the Internet to a secure server. If necessary, case managers interact with intervention patients for medication adjustments (up / down) via a web portal using a data transmission protocol known as Health Level 7 (HL-7; www.hl7.org). Control patients will take their BP recordings to their primary care physician (PCP) or nurse practitioner (NP) during their routine check-up visits.

Due to the ongoing COVID-19 restrictions for face-to-face patient contact, we will train patients through phone and video contact in addition to sending each participant a written information outlining the study procedures, how to measure their BP, how to download and use the Sphygmo App, and how to transmit BP measurements from their smartphones (Supplementary Appendix 1 and 2). Thus, we will train patients to measure BP using guideline recommendations^10^ leveraging a locally developed and validated HBPT system (http://mmhg.ca/about-us/). Patients will receive a Bluetooth-enabled and validated electronic upper arm oscillometric BP device (A&D Ltd. UA-651BLE; San Jose, California) that will be paired to their smartphone. Instructions on how to measure appropriate cuff size will also be provided to each patient (Supplementary Appendix 2). Patients will be required to sit with their back rested for at least 5 minutes with the BP cuff around their arm. They will then be required to push the start button on the HBPT device to initiate BP measurement. HBPT values will be based on a series comprised of the mean of duplicate measures, for morning and evening, for a 7-day period and the first day home BP values will not be considered.^11^ Thus, mean SBP for the week will be computed from average of duplicate readings for the next 6 days, that is, 24 measurements. The BP data will be auto transmitted via Bluetooth to their smartphone and relayed to a secure web portal for review (Figure 3). This 7-day protocol will be repeated each month until BP is in the required target range. Once BP is controlled, that is, guideline-concordant, the 7-day protocol will be repeated every 3 months for 1 year. The smartphone platform is already established in 4 markets (Canada, United States, Hong Kong, Singapore) and is widely used by members of the target population^29^ and a similar system has been tested in a pilot study of 20 patients from the University of Alberta’s Hypertension Clinic as well as in a trial of BP telemonitoring in very elderly patients residing in supportive living environments.^30^

**Figure 4.**
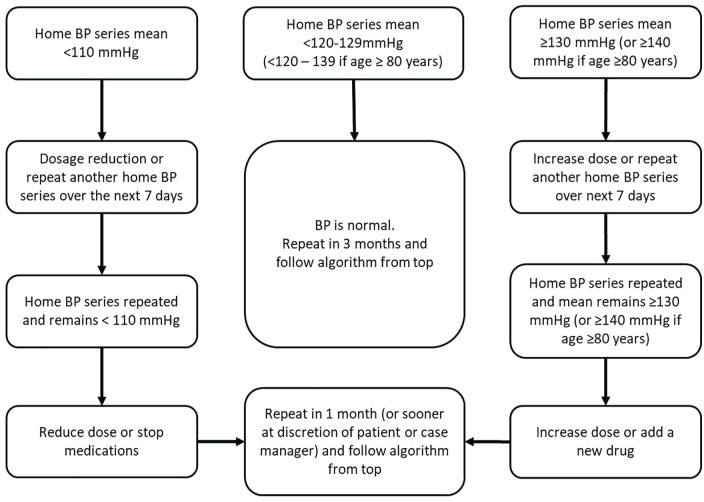
Case manager protocol for BP lowering medications. *Note.* A home BP series will consist of a 24-reading average (duplicates measured in the morning and evening) taken over a consecutive 7-day period. Medications will be added in a guideline-concordant manner. We will ensure that all patients are at least on an Angiotensin converting enzyme-inhibitor (ACE-i) and diuretic, unless medically contraindicated. BP = blood pressure.

Tele-transmitted BP readings will be summarized within the server using telemonitoring software and temporal trends will be plotted. For the active arm of the study, BP data will be tele-transmitted to the study case manager, who will oversee lifestyle modifications and BP self-monitoring and medication adherence. The case manager will also review telemonitored BP summaries, make protocol-based therapeutic adjustments based on a defined algorithm (Figure 4), and send summaries to participants’ PCPs to inform them of treatment changes to facilitate communication between patients and care providers. Medications will be adjusted based on guideline recommendations^10,11^ and existing treatment protocols used in other chronic disease settings (ie, diabetes and stroke).^31^ Participants will also receive a document that shows how to unlink their account and delete the Syphmo App from their smartphones at the end of the study (Supplementary Appendix 3).

#### Control arm

Patients in the control arm (usual care) will receive the same HBPT device and BP measurement training as those in the intervention arm and will also follow the same measurement protocol—That is, 2 measurements taken in the morning and 2 taken again in the evening for 7 consecutive days. The values from the first day will be discarded and the mean SBP for the week will be computed from the average of the readings from the following 6 days, that is, 24 measurements. However, the study case manager will not interact with patients in this group. Patients in his group will be expected to inform their PCP or nurse practitioner (NP) of their BP readings at their scheduled consultations. For those in this group whose 6-day average SBP readings are ≥ 220/110 mmHg or <70 mmHg, the case manager will notify the Data Safety and Monitoring Committee (DSMC) for urgent review, treatment plan and referral strategy. Each participant’s PCP / NP will be informed of the patient’s involvement in the trial and will receive a letter with a copy of the study synopsis and 1-page guideline summary for BP thresholds, targets, and treatments relevant for CKD.

### Study Procedures and Data Collection

At baseline, relevant demographic and health behavior information including age, sex, race, smoking, alcohol intake and details of patient’s medical history such as significant CV disease (eg, coronary artery disease, stroke, peripheral arterial disease, heart failure) and medication history (BP-lowering drugs—name, type, dosage, frequency, and duration) will be collected. Relevant clinical details such as body mass index (BMI) will also be recorded. Blood pressures will be measured and recorded following guideline recommendations.^10,11^ Other information that will be collected includes health care use in the past year (physician and/or emergency room visits, and hospitalizations) ascertained through patient self-reports and/or provincial administrative data sources, quality of life (Kidney Disease Quality of Life-36 [KDQOL-36]) and utility measurements (European Quality of life Five Dimension [EQ-5D]); and satisfaction with receiving health care, using the Patient Assessment of Care for Chronic Conditions (PACIC-2.0).^29-32^

Laboratory measurements will be carried out at baseline including serum electrolytes, serum creatinine (and estimated glomerular filtration rate), glycated hemoglobin (HbA1c), and urinary albumin/creatinine ratio (UACR). These measurements will also be taken at 6-months and 1 year.

Assessment of usability and acceptability is critical for all technology-enhanced care interventions to minimize risk of undesired consequences commonly seen post-implementation.^33,34^ End-user input into system design and operation is important throughout the evaluation process to reduce the risk that interventions will be ineffective, unusable, or unsafe. Usability testing will involve assessment of human-computer interaction—specifically, issues related to use, interface, design, function and will also include acceptability of the model of care for the caregivers. We will take a qualitative approach to this process by using focus groups. We plan to recruit 2 focus groups, 1 for patients in the telemonitoring arm and 1 for care providers (case managers and PCPs), with a target sample size of 5 to 8 participants in each group. The first 5 users typically identify 70% of severe usability problems and the first 8 users typically identify 85%; a higher sample size has low incremental yield due to data saturation.^35^ Prior to setting up the patient focus group, an advisory group consisting of patients with CKD and hypertension will first be asked to review contents of the focus group questions for relevance and applicability to real-life patients with hypertension and CKD. Focus group questions for the PCPs will be jointly developed by qualitative research experts and the investigators.

Focus groups will be conducted separately for each of the groups (patients and care providers) after 3 months in the study and again before the end of the study (Figure 2). Sessions will be audiotaped, transcribed verbatim and analyzed using the methodology of interpretive description (ID).^36,37^ ID is a methodology developed to generate knowledge about general patterns and themes that could then be applied to inform care for individual patients.^38^ Well-established methods will be used to ensure trustworthiness and rigor, including credibility (iterative cycles of engagement), confirmability (audit trails) and transferability (reporting on kidney disease in the rural context).^37,39^ NVivo software application will be used to code and manage qualitative data and to create a filing system and coding database. Transcripts from the first focus group session for each group will be read and re-read to generate an initial codebook. The codebook will be iteratively refined throughout the analysis. Codes will be categorized and analyzed thematically.^37^ Patient and clinician data will first be analyzed separately, and then across groups. Patients’ responses regarding usability and acceptability will be analyzed further in relation to care for their BP control. All groups will offer feedback on acceptability of the system and suggestions for improvement for use in patient care.

Trial-based costing will be assessed with the 3-step micro-costing technique of identification, measurement and valuation of relevant health care and non-health care resources using standard methods,^40,41^ with a focus on cost of telemonitoring and case management, health care costs (through linkage with Alberta Health data), and patient-borne costs (patient survey). Thus, the cost-effectiveness (Model A) and cost utility (Model B) of the intervention will be compared against the control using a modified version of a validated economic model used by the team in prior work on HBPT and case management^42^ and other chronic diseases.^41-43^ Best practices for economic evaluation will be followed.^41,44-46^ The intervention includes intermittent or one-time costs (eg, devices, treatment algorithm development, patient and health care professional training) and ongoing costs (eg, network and data, case manager time, BP medications). Data captured during the RCT will accurately determine the costs of telemonitoring, case management, and health care use by patients (physician, ER visits, etc.). The change in BP at 1 year, a validated surrogate, will be a key variable in both analyses:

- Model A will calculate the cost per decrement in mean BP using a 1-year time horizon and a public health care payer perspective.
- Model B will estimate incremental cost per quality-adjusted life year (QALY) gained over a lifetime.^42,47^ It will consider longer-term health outcomes, including the probability of developing end-stage kidney disease (ESKD) (requiring dialysis or transplant) or major CV events (ie, coronary artery disease, heart failure, stroke), all-cause mortality, impacts on quality of life (EQ-5D), and health care costs associated with these outcomes.

In each model, we will explore distributions using bootstrapping, create acceptability curves, and perform one-way and probabilistic sensitivity analyses to explore the impacts of uncertainty in key model parameters. In Model B, the potential impact of reducing complications of treating hypertension in patients with CKD (hypotension, syncope) by a plausible range will be explored in a sensitivity analysis using baseline risk determined from a population-based cohort of patients^48^ and ambulatory care sensitive conditions^49^ for emergency room visits and hospitalizations.

### Outcomes

The primary outcome will be the mean difference (MD) in home SBP at 12 months, from baseline values. Secondary outcomes will include (1) proportion of patients with SBP within guideline target at end of the study, from baseline; (2) Proportion of patients who remained within target SBP throughout the study; (3) user acceptability of HBPT for BP control; (4) cost-effectiveness and cost utility of HBPT combined with a protocol-based case management approach, and (5) adverse events reported.

### Adverse Events and Safety Monitoring

An independent DSMC will monitor the trial for safety and efficacy. Safety concerns that will apply to this study will include any syncopal event, hypotension (SBP <110 mmHg requiring medical assistance), occurrence of severe hypertension (SBP ≥ 220 mmHg and/or DBP ≥ 110 mmHg), hyperkalaemia (serum potassium ≥ 6.5 mmol/L), and worsening kidney function (doubling of serum creatinine or 50% reduction in eGFR from baseline value). Safety concerns will be reviewed immediately by the case managers and appropriate referral, or management instituted. The DSMC will review the data regularly or as soon as cases are referred to them by the investigators and will determine if patients should continue their participation in the study.

### Sample Size Estimation

The sample size calculation for this trial is based on the data from HBPT plus pharmacist management trial on the hypertension population.^22^ Adequate power will be used to detect a clinically important absolute difference in mean SBP of 10 mmHg between the intervention arm and the control arm (usual care). Using a 2-sample t-test, alpha of 0.05 and power of 0.80, and assuming a common standard deviation of 19 mmHg, the required sample size will be ~58 patients per arm, or 116 in total. Accounting for ~20% attrition over 1 year, we will recruit 73 patients per arm, for a total of 146 patients. This sample size is also powered to detect a difference of 20% in the proportion of patients who maintain the guideline target of SBP (alpha 0.05, power 80%, and assuming event occurs 50% in the control group).^22^

### Plan for Data and Statistical Analysis

Baseline statistics and the outcome variables will be described using counts and proportions, mean (SD) or median (interquartile range) as appropriate. All analyses will follow the intention-to-treat principle. We will estimate the effect size as the mean difference of SBP between groups at 12 months using a mixed linear-regression model include fixed-effects term for time point (6 months and 12 months), intervention, their interaction, and a random-effect term for participants. For the dichotomous outcomes (patients who maintained the SBP guideline and adverse outcomes), we will use Poisson regression with robust error variance to test the effect of the intervention.^50^ Continuous outcomes without repeated measurement will be examined using linear regression. We will adjust for eGFR for all the analyses. All estimated effect sizes will be reported along with 95% confidence intervals, and P<0.05 was considered statistically significant. We will analyze the data using Stata/MP 17.0 software (www.stata.com).

We will use multiple imputation method for missing data. Imputation of missing SBP values will only apply in instances when participants do not have any blood pressure recordings for the entire week. We will impute the missing SBP within each randomized group using age and sex separately at 6 months and 12 months. Estimate from each imputed data set will be combined using Rubin’s method.^51^ No interim analyses are planned due to the short duration of the study. However, for subgroup analysis, we will use the model from the analysis for primary analysis to explore the relationship between intervention and eGFR groups, age groups, the presence of diabetes, and so on.

### Data Monitoring and Quality Assurance

Data will be entered directly at the site of data collection using laptops and an online REDCap database application. REDCap uses a secure syncing process and robust data validation techniques. The statistician will create REDCap reports that tabulate accrual, withdrawals, fully completed protocols numbers and create quality assurance queries. The study coordinator will run these reports and queries and then follow-up with the pertinent study personnel or participant.

### Ethics and Privacy Statement

Ethics approval for this study has been obtained from the University of Alberta Research Ethics Committee (#PRO00095231). To ensure patient confidentiality and that all collection, retention, use or disclosure of data complies with privacy legislation and ethical requirements, all personal identifiers will be removed from the research data repository.

### Integrated Knowledge Translation Strategy

TIKO is a collaborative project among researchers, knowledge users, and patient stakeholders. Relevant stakeholders have been involved from the project’s inception to ensure that it addresses the needs of patients and practitioners. We will provide quarterly updates tailored to each of the stakeholder groups (ie, policy briefs and fact sheets for policymakers, and infographics containing key messages for patients and PCPs). Our patient partners will contribute feedback that will inform the implementation of the intervention and will be instrumental in disseminating study updates and results to patients, care providers, and policymakers. To ensure the support required for patient and other stakeholder collaboration in this work, we will leverage the resources available from the Alberta SPOR Network. Adherence to the guiding principles for patient engagement in health research (inclusiveness, support, mutual respect and team building) will ensure that participation is mutually beneficial for all members of our research team.^52^

## Discussion

The key deliverable of this proposal is the generation of evidence that would allow targeted and effective population-level strategies to be implemented to improve health outcomes for high-risk hypertensive CKD patients in Canada’s rural and remote communities. Specifically, this work will: (a) reduce under-treatment, thereby slowing progression to ESKD and preventing CV disease; (b) reduce over-treatment and associated adverse consequences (eg, syncope, falls, worsening renal function); and (c) demonstrate proof-of-concept and provide the basis for developing relevant policies and knowledge translation strategies to enhance the uptake of findings in chronic disease contexts beyond CKD. Our findings will inform guidelines specific to the management of CKD and support policy recommendations that can be scaled to other high-risk population groups and chronic disease domains.

**Figure 5.**
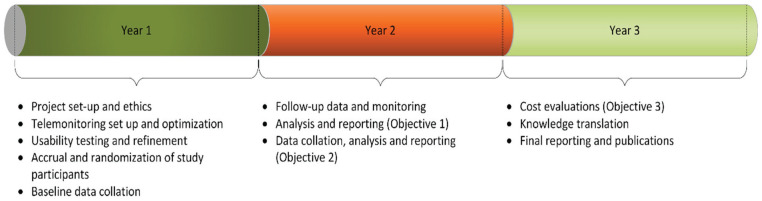
Timeline and milestones.

This proposal focuses on a high-risk population subgroup characterized by a large care gap and identified by PCPs as a priority—namely, patients with CKD and uncontrolled BP. The proposed intervention technology is eminently simple and feasible, and it represents a low-risk endeavor with high potential for a good return on investment. Moreover, leveraging on a team that includes all key players (patients, decision makers, PCPs and researchers) involved in the design and implementation of a pragmatic intervention further improves the feasibility of this study. The involvement of relevant clinical program leaders underscores the potential for broad implementation of key findings, policy partner buy-in, and scalability. Within the first 6 months, we will hire personnel, develop the case report forms, set up the telemonitoring process and web portal, perform on-site pilot testing and refine the processes. Patient recruitment will begin after 6 months and timelines to meet all study objectives are shown in Figure 5.

### Potential Challenges and Mitigation Strategies

We have identified potential challenges to this study and have prepared mitigation strategies to address them. First, although the 1-year follow-up period could be considered short, however, longer follow-up period is often desirable when observing hard outcomes (eg, progression to dialysis, time to first / repeat CV event, death, etc.). This follow-up duration was selected as we recognize that BP is a surrogate outcome and is the most important measure for predicting adverse CKD outcomes. Also, it is well established that reducing high BP among patients with CKD substantially reduces complications and risks of adverse events. Avoiding low BP and resultant adverse effects is also important. However, beyond the scope of this study, we will examine how treatment and monitoring affect hospitalization rates, CV events, and mortality over long term. Second, possible technological pitfalls may provide challenges to conducting this study. We note that frequent monitoring and intervening too aggressively may also cause adverse consequences. Such consequences will be minimized by responding to qualitative feedback, standardizing telemonitoring frequency, ensuring careful monitoring for adverse effects and changing drug regimens as necessary. Third, owning a smartphone and having access to wireless internet is an eligibility requirement for this study and could therefore exclude potentially eligible patients who do not have smartphones. However, as more than 85% of Canadians own a smartphone^53^ and have access to the internet, we do not expect that this will constitute a major barrier with recruiting participants in this study. However, it is anticipated that this proportion could be lower in the remote setting of our study and could provide a challenge to the execution of this study. In such instances, the study team will provide a smartphone and internet access to such patients. Fourth, poor access to medications, reluctance to titrate mediations despite telemonitoring and access to urgent care could also prove challenging. However, the steps we have highlighted for reviewing transmitted BP recordings and steps for referral should mitigate these challenges. Finally, operational costs could provide challenges and similar trials have proven expensive because of the cost of home BP monitors. However, we will minimize cost through use of simple, inexpensive devices and focusing on data relevant to the study’s objectives.

## Supplemental Material

sj-pdf-1-cjk-10.1177_20543581221077500 – Supplemental material for Telemonitoring and Case Management for Hypertensive and Remote-Dwelling Patients With Chronic Kidney Disease—The Telemonitoring for Improved Kidney Outcomes Study (TIKO): A Clinical Research Protocol Click here for additional data file. Supplemental material, sj-pdf-1-cjk-10.1177_20543581221077500 for Telemonitoring and Case Management for Hypertensive and Remote-Dwelling Patients With Chronic Kidney Disease—The Telemonitoring for Improved Kidney Outcomes Study (TIKO): A Clinical Research Protocol by Ikechi G. Okpechi, Deenaz Zaidi, Feng Ye, Miriam Fradette, Kara Schick-Makaroff, Charlotte Berendonk, Abdullah Abdulrahman, Branko Braam, Anukul Ghimire, Vinash Kumar Hariramani, Kailash Jindal, Maryam Khan, Scott Klarenbach, Shezel Muneer, Jennifer Ringrose, Nairne Scott-Douglas, Soroush Shojai, Dan Slabu, Naima Sultana, Mohammed M. Tinwala, Stephanie Thompson, Raj Padwal and Aminu K. Bello in Canadian Journal of Kidney Health and Disease
