# Supplementary material for: Telemonitoring and Case Management for Hypertensive and Remote-Dwelling Patients With Chronic Kidney Disease—The Telemonitoring for Improved Kidney Outcomes Study (TIKO): A Clinical Research Protocol
Source: Can J Kidney Health Dis. 2022 Feb 13;9:20543581221077500. doi: 10.1177/20543581221077500 (PMC8848092; doi:10.1177/20543581221077500)
Supplement: sj-pdf-1-cjk-10.1177_20543581221077500 – Supplemental material for Telemonitoring and Case Management for Hypertensive and Remote-Dwelling Patients With Chronic Kidney Disease—The Telemonitoring for Improved Kidney Outcomes Study (TIKO): A Clinical Research Protocol [file sj-pdf-1-cjk-10.1177_20543581221077500.pdf]

SUPPLEMENTARY MATERIALS

Supplementary Appendix 1:

How to measure your blood pressure

(Figure adapted from: https://kdigo.org/wp-content/uploads/2021/03/KDIGO-BP-Guideline-Measurement-Figure.pdf)

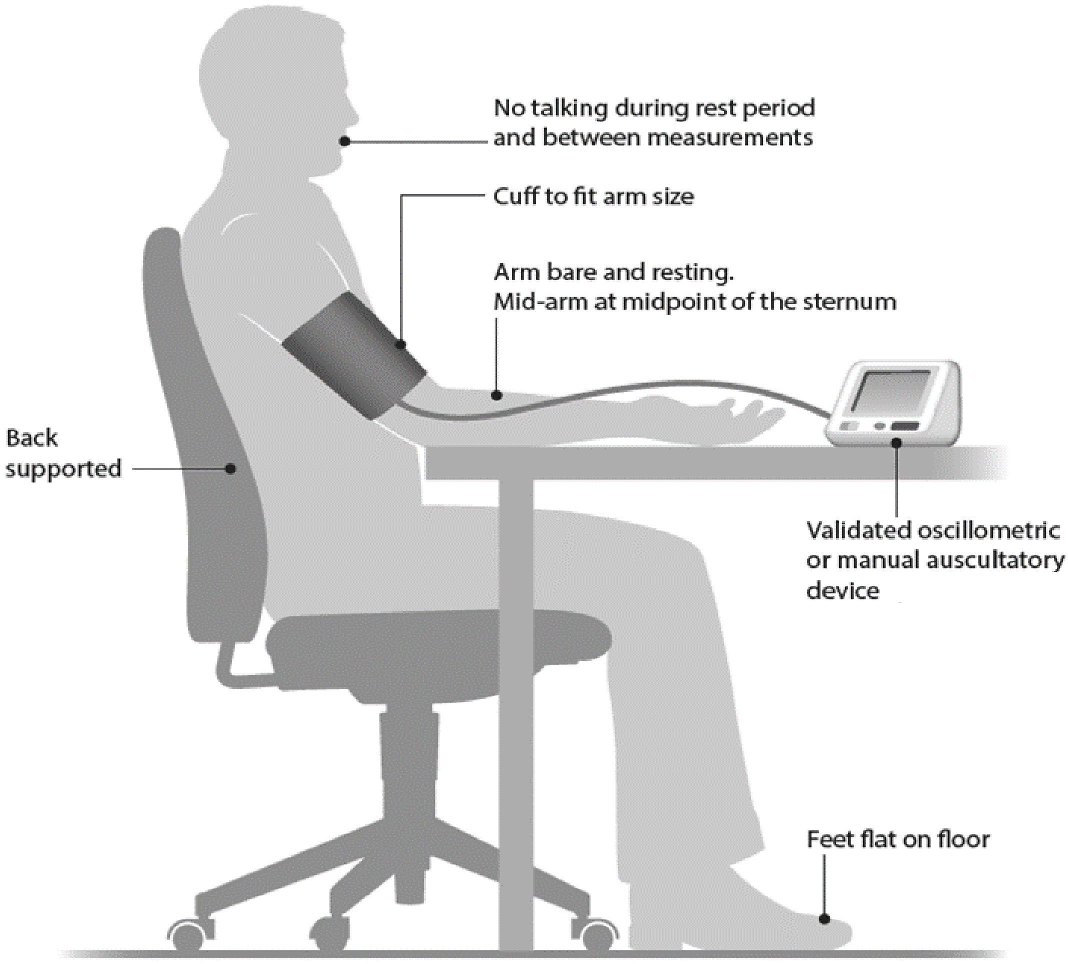

- Take the measurement in a quiet room (no talking during rest period between measurements)
- No smoking, caffeine, or exercise for 30 min before measurement
- Empty bladder
- Sit relaxed with back supported for >5 min before commencing measurements
- Ensure the BP cuff covers at least 80% of the mid-arm circumference
- Separate repeated measurements by 1–2 minutes

Supplementary Appendix 2

How to download and use the Sphygmo App

Step 1:
Download the free smartphone app '**Sphygmo BP and Glucose**' available on Apple App Store or Google Play. It is offered in English, Spanish, French and Chinese (according to the user's smartphone language setting).

Step 2:
Register an account using an email address and password and accept the terms of use.

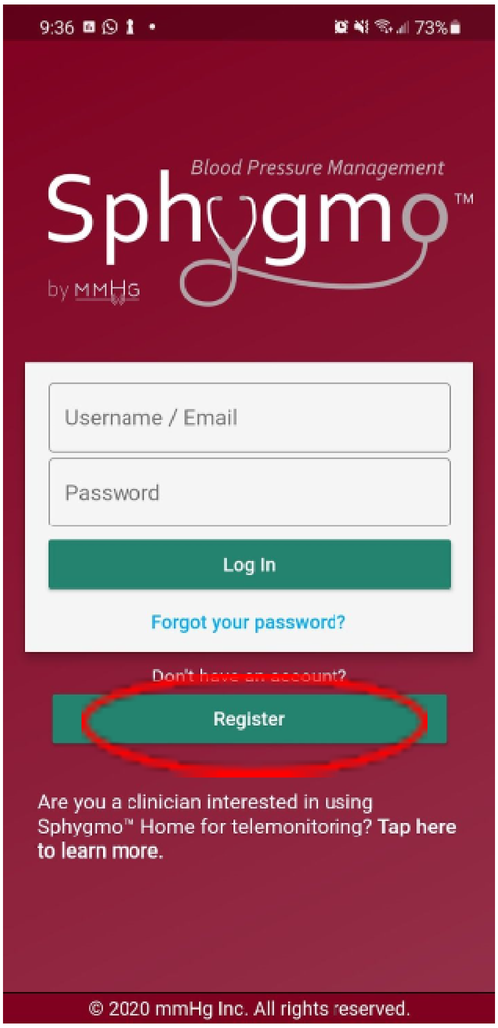

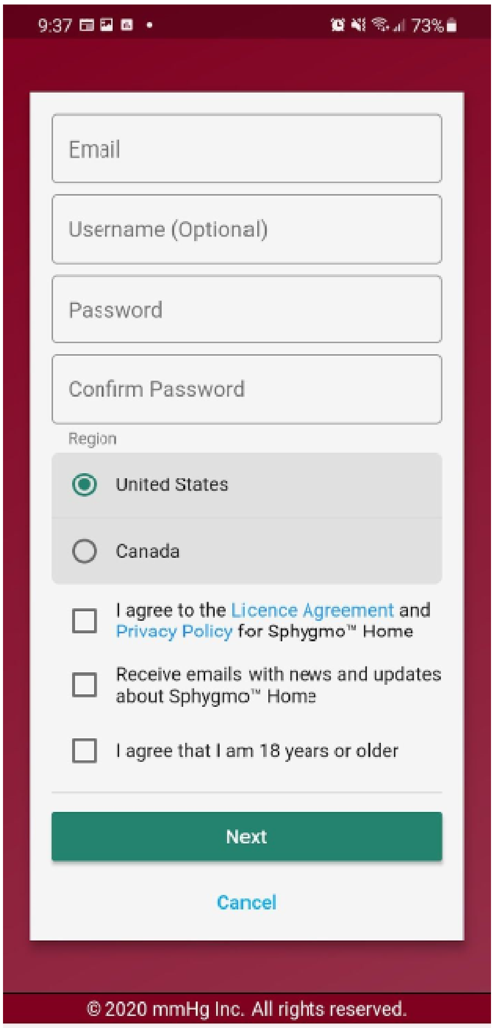

Step 3: You will be asked to enter a link code. Please enter **TIKO**. Then, put in your name, date of birth, and sex (so that the TIKO study team can identify you).

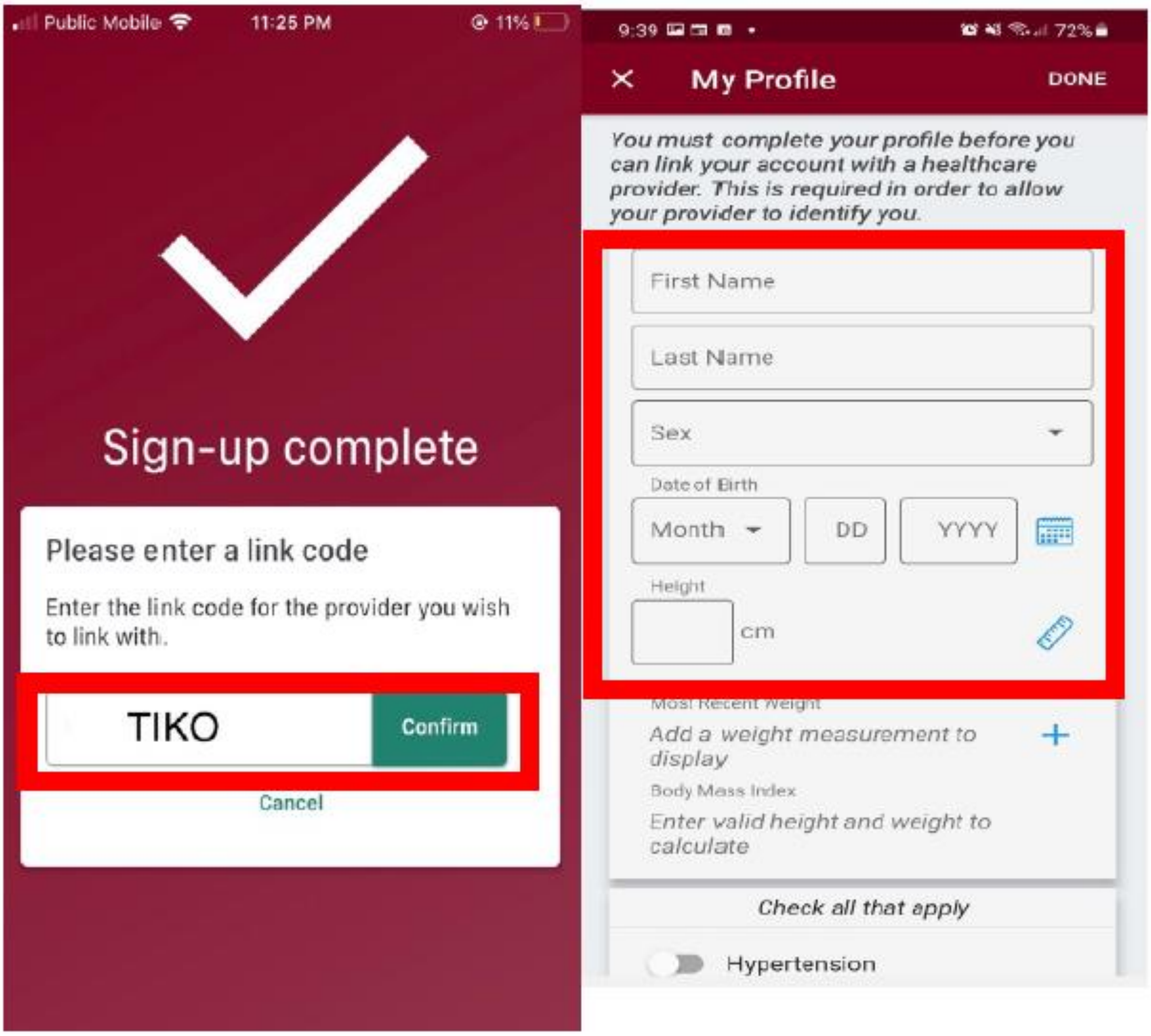

You can also link your account through the home page:

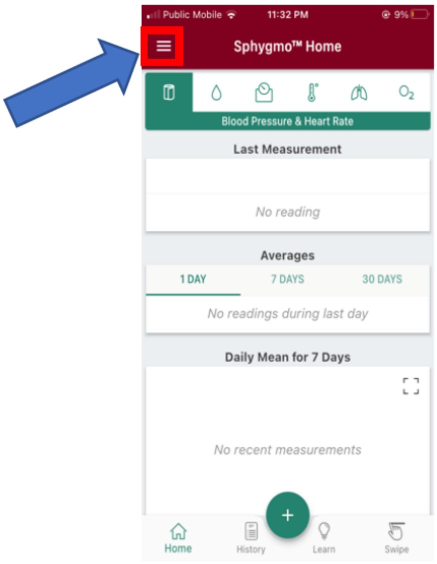

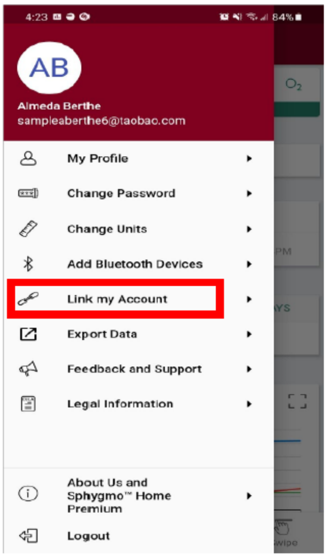

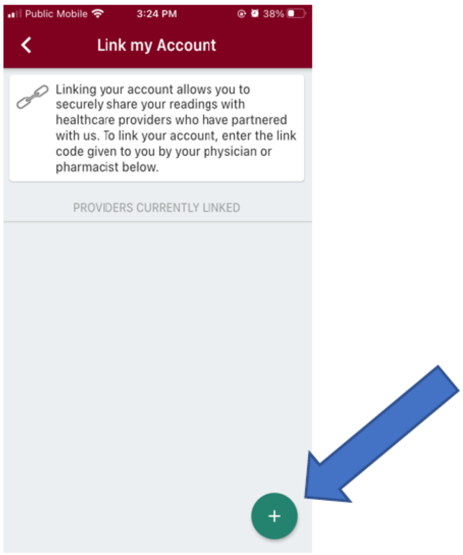

1. Select hamburger menu 2. C l i ck ‘Link my Account’ 3. Select green ‘+’ button

Step 4: Pair your Bluetooth blood pressure monitor (steps below)

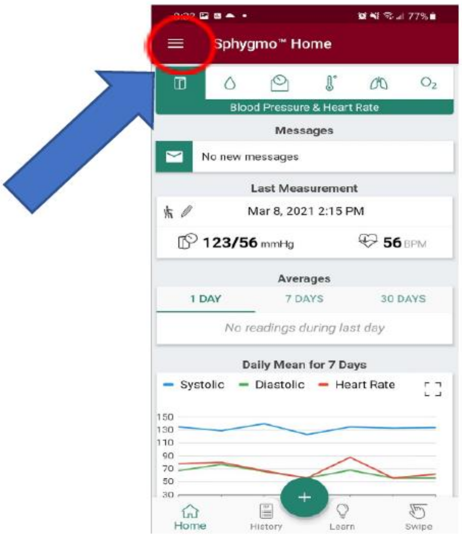

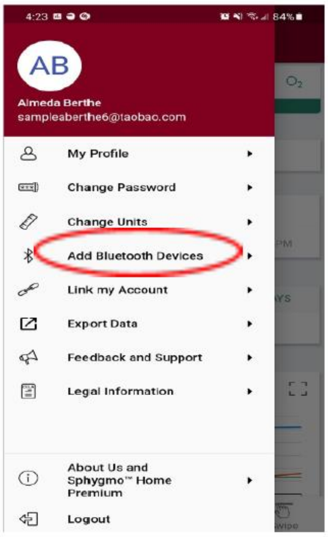

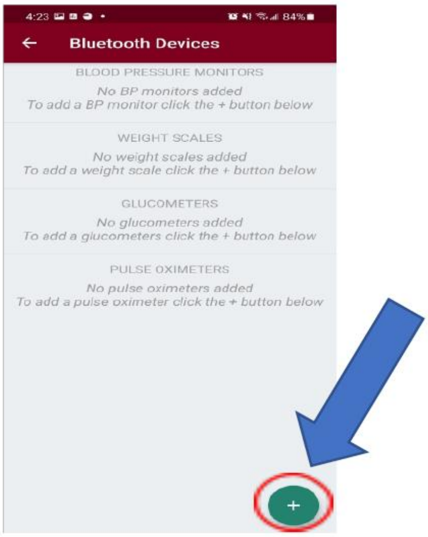

1. Select hamburger menu 2. Select ‘Add Bluetooth Device’ 3. Select green ‘+’ button

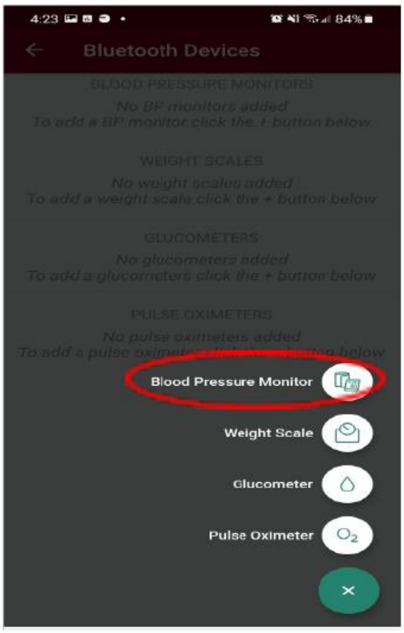

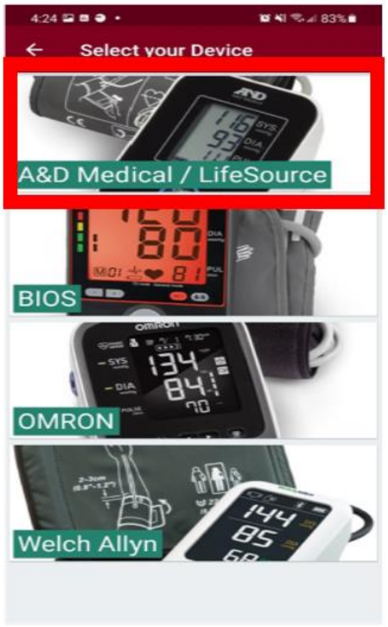

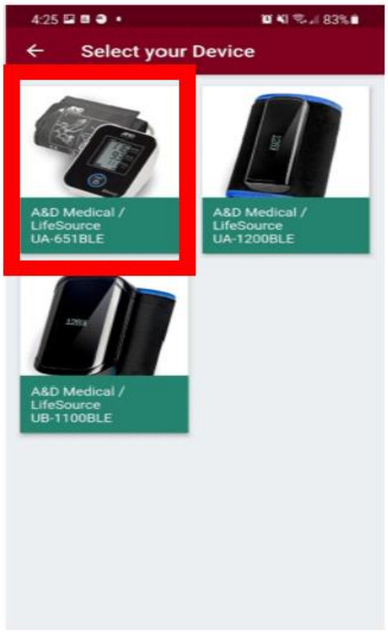

4. Select ‘Blood Pressure Monitor’ 5. Select ‘A&D Medical / LifeSource’ model 6. Select device

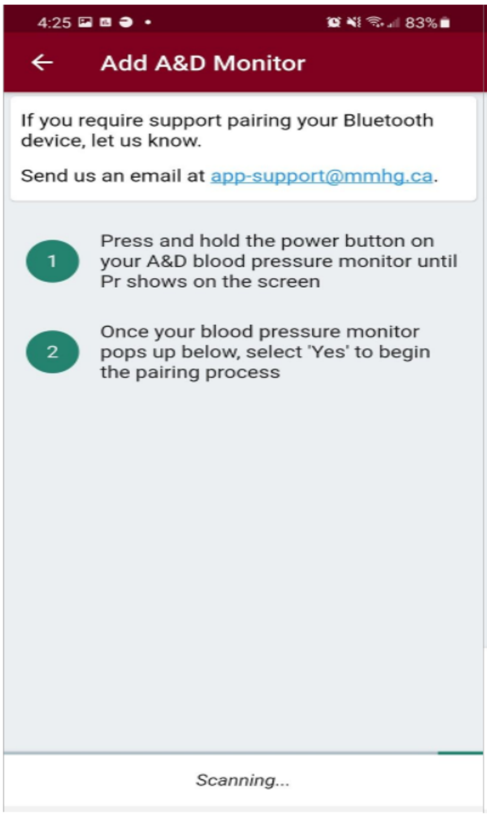

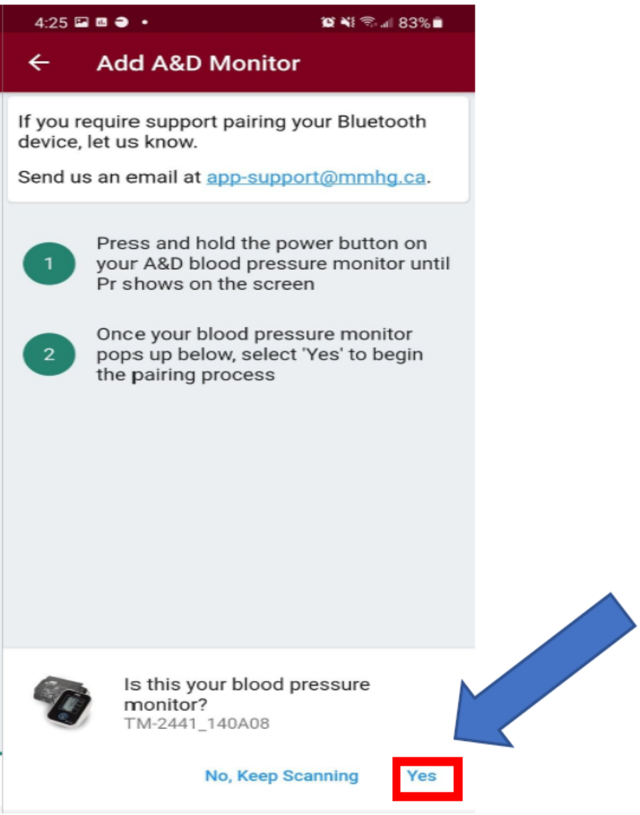

7. Follow the pairing steps 8. Select ‘Yes’ for initiated device

Sphygmo will now send your blood pressure measurements to the TIKO study team.

How to check your first Blood Pressure measurement

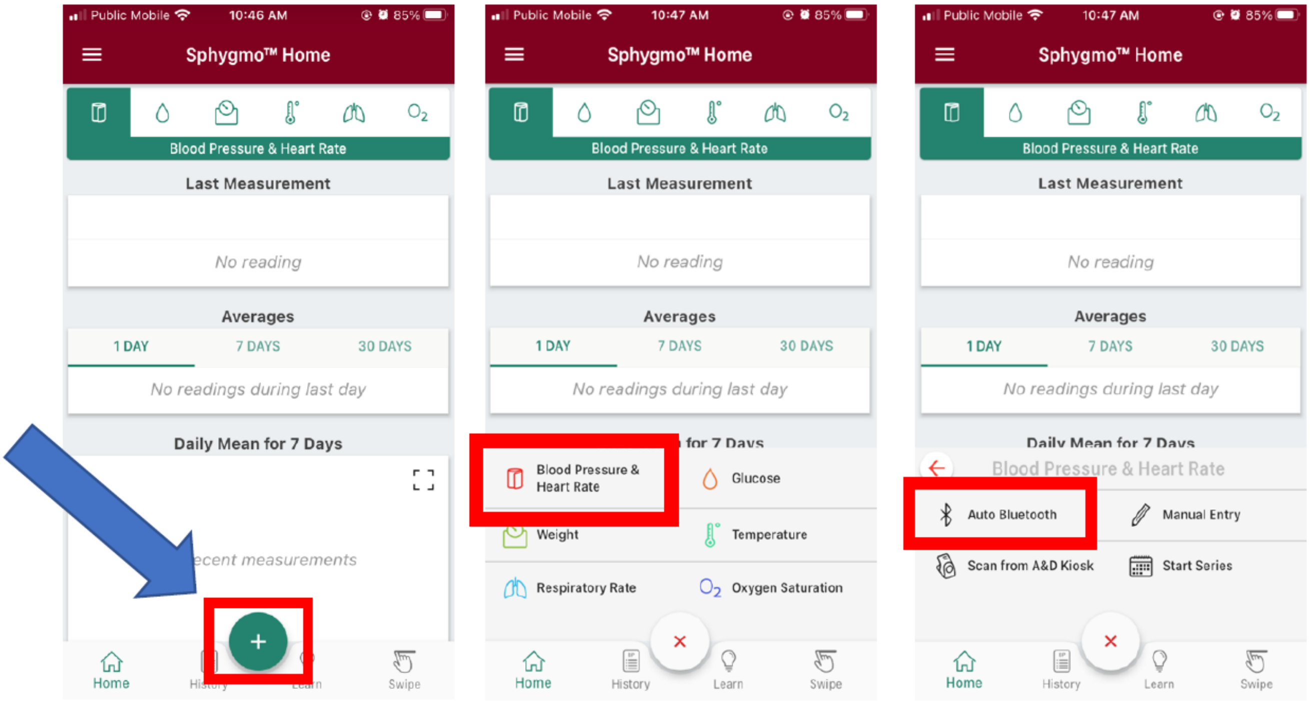

1. Go to the home page and click on the green '+' button at the bottom. 2. Select 'Blood Pressure & Heart Rate' 3. Select 'Auto Bluetooth'

Auto Bluetooth will allow the transmission of your readings from the BP machine to your device. Please ensure that you have paired your device to the machine through Bluetooth (steps on the previous page), then follow the steps below:

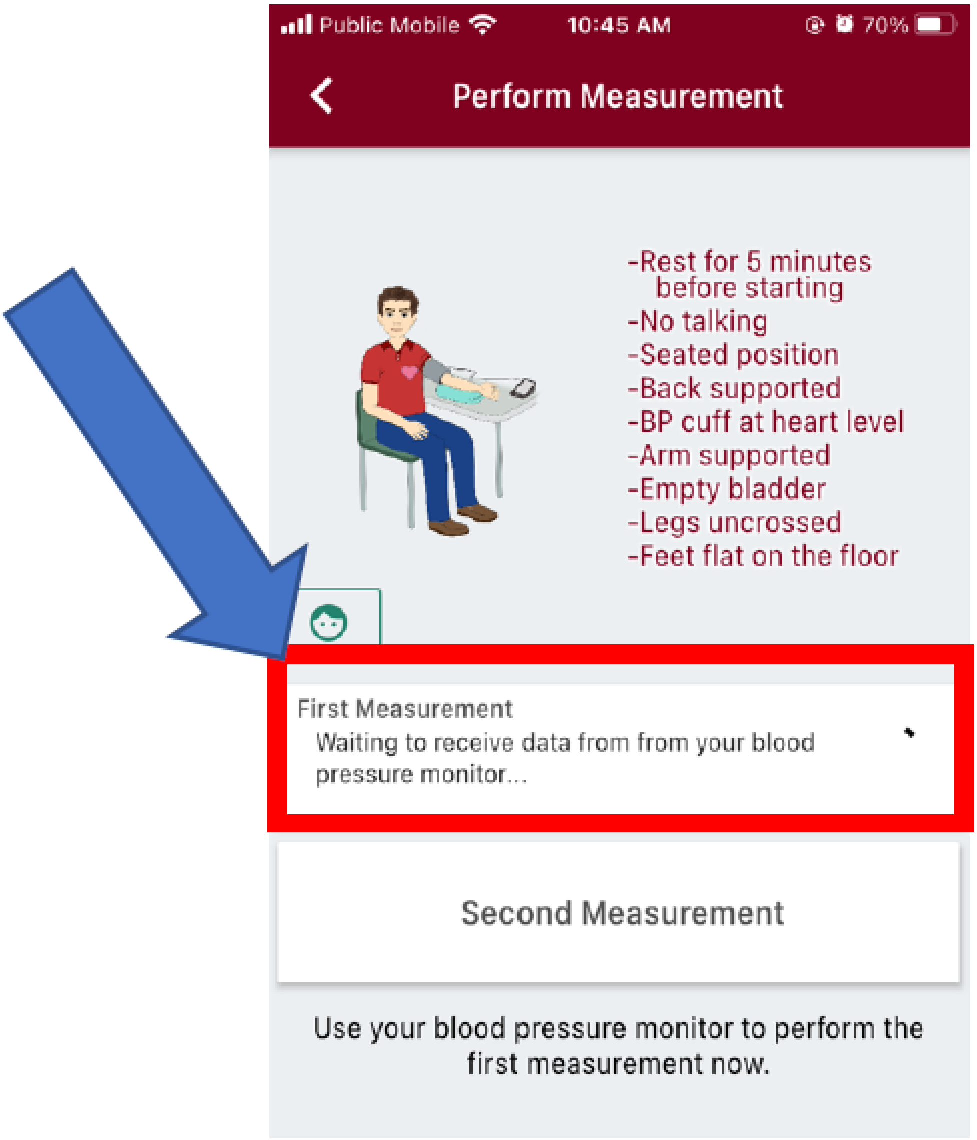

1. Begin your first measurement simply by turning on your BP monitor.

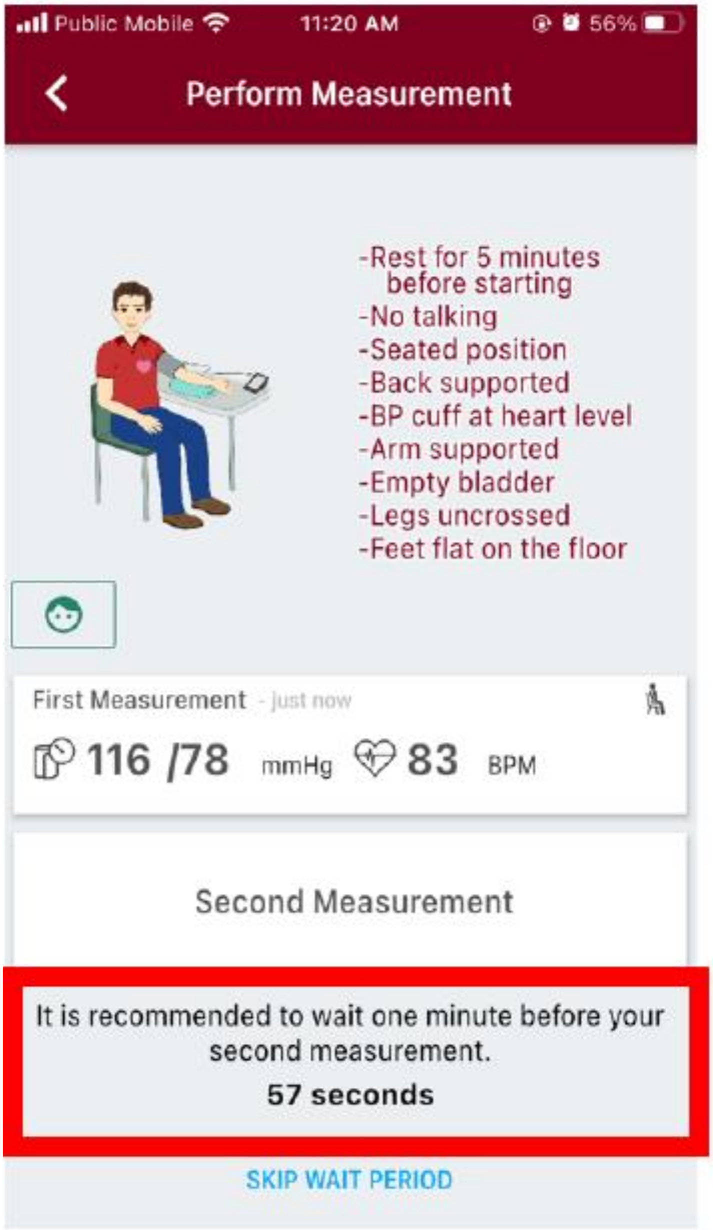

2. After your first measurement, you will be asked to wait one minute before taking your second measurement.

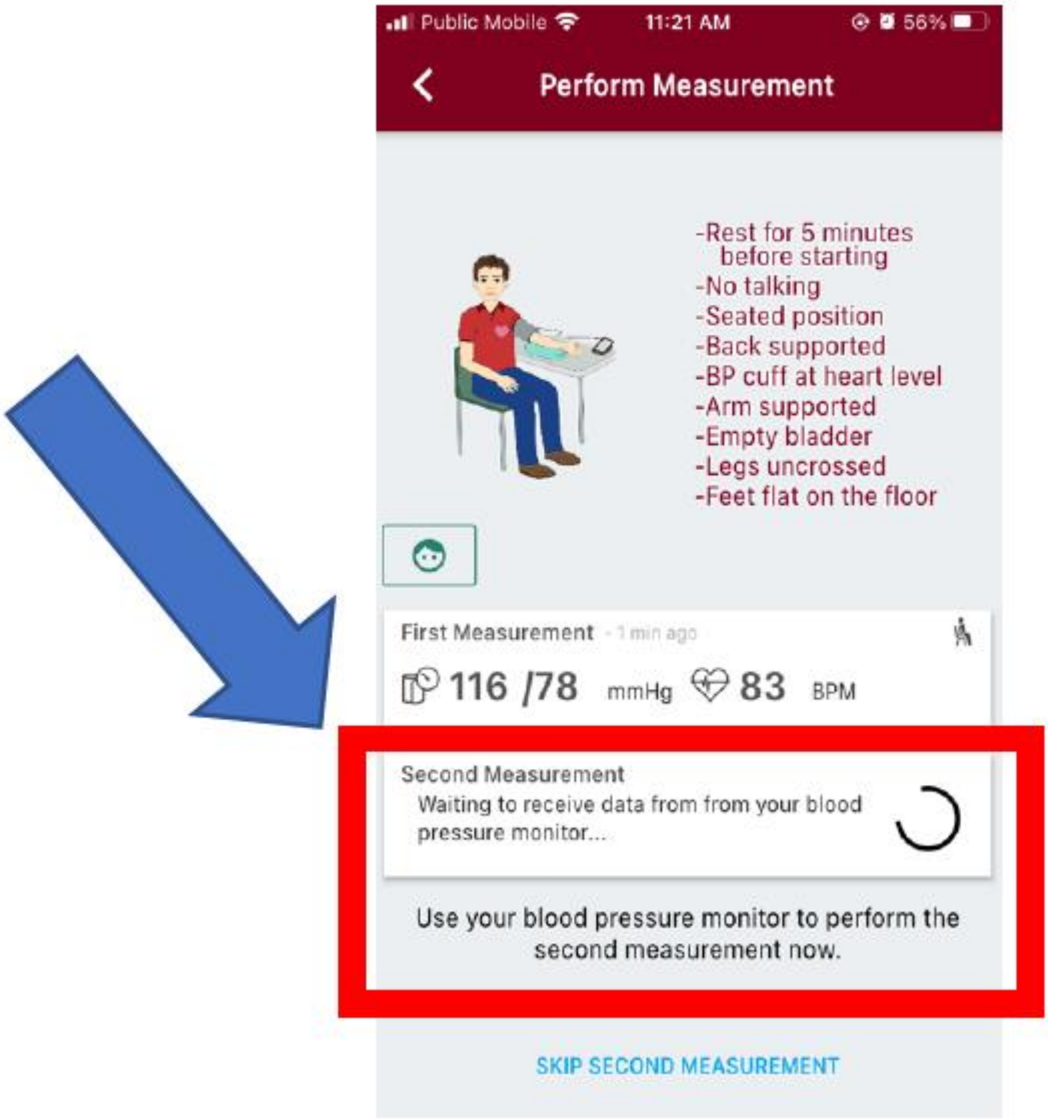

3. After the minute mark, you will be prompted to take your second measurement, by turning your BP monitor on again

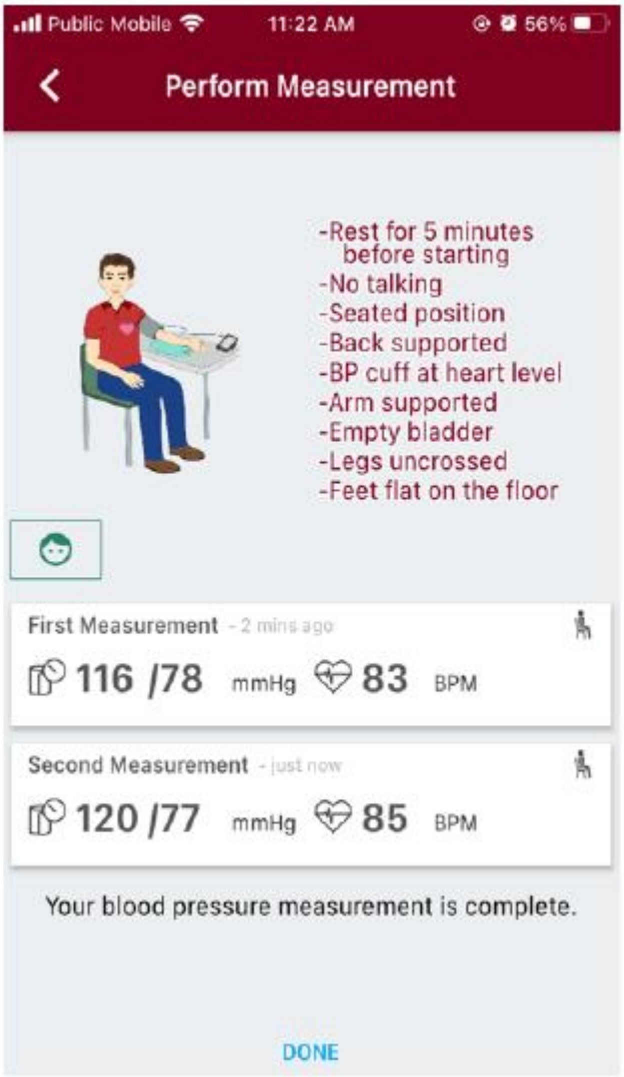

4. Once both readings are complete, you may exit the page. Continue these steps for your next few readings.

App Navigation and Features

<u>Hamburger menu. Click here to:</u>

- View your profile settings
- Change your password
- Connect your monitor through Bluetooth
- Link your account

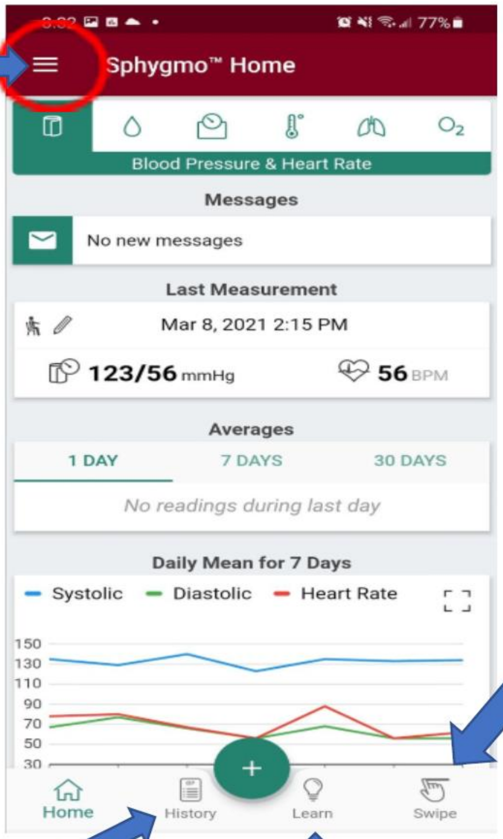

<u>Swipe-Averaging Feature. Click here to:</u>

- View a summarization of your blood pressure measurements
- Compare measurements between different days/weeks

Click here to view your past readings

<u>Click here for more information on:</u>

- How to choose a blood pressure monitor
- High blood pressure (hypertension)
- Home Blood Pressure Monitoring
- Reducing your blood pressure
- Low blood pressure (hypotension)

Measuring Arm Circumference

Measuring your arm circumference is important as it will determine your cuff size. Please note the following when doing the measurements:

- Have **someone else** take the measurements on your arm, while you are standing with your back to them
- Expose your **entire arm**
- Measure the arm that you are using for your blood pressure device

Please see instructions below:

Part 1: Marking the Arm

1. Position your arm so that it is flexed 90° at the elbow, with your palm facing up

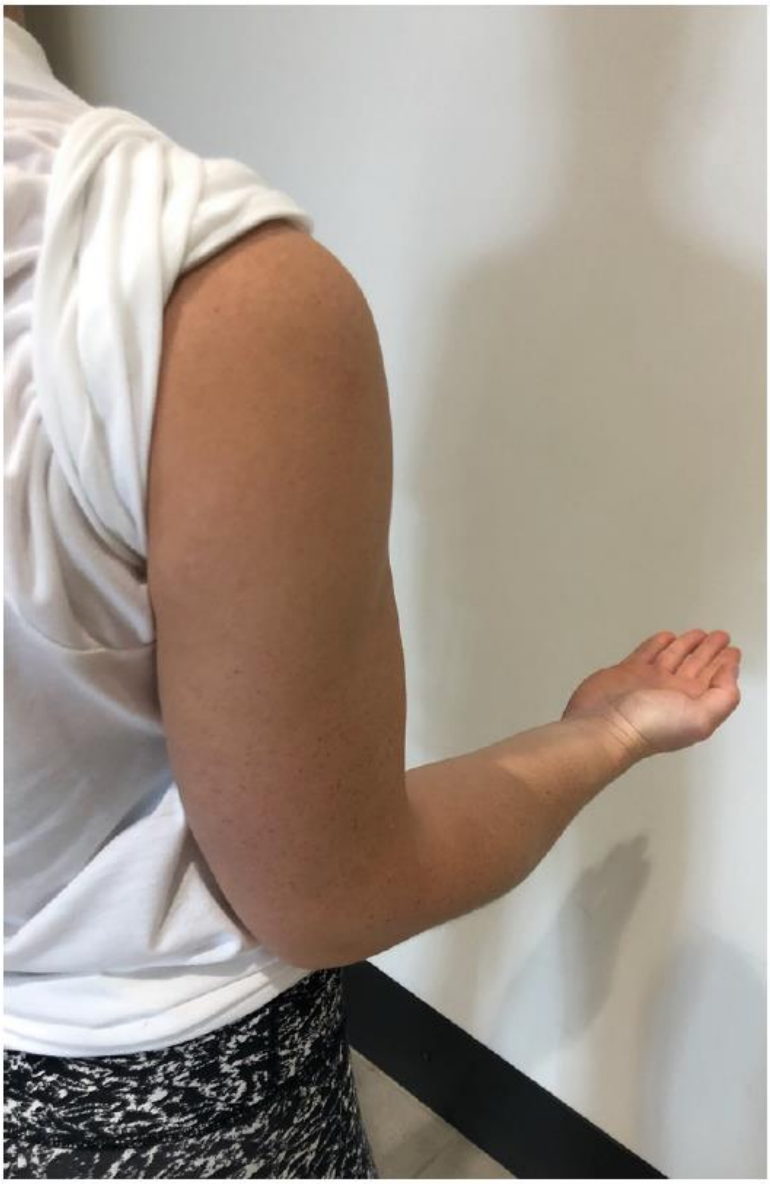

2. Find the tip of the shoulder and mark it with a cross

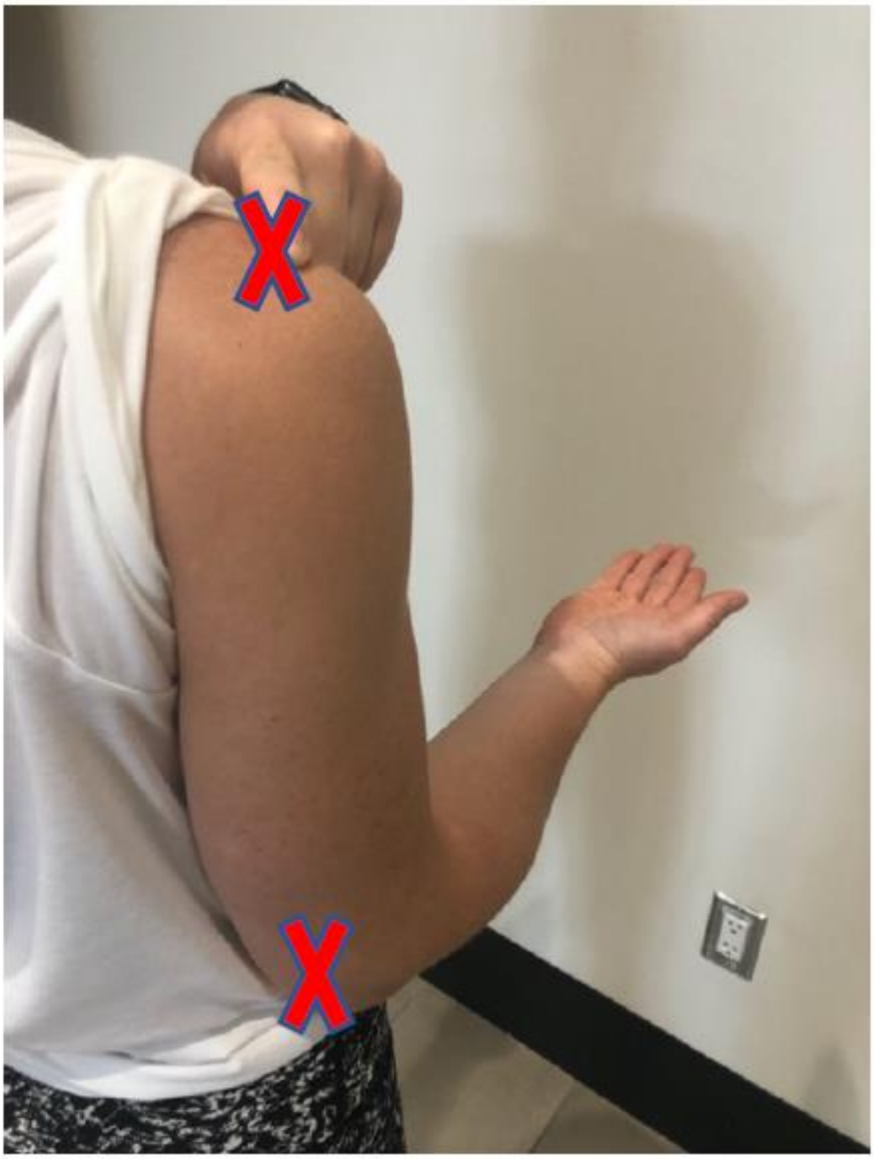

3. Find the tip of the elbow and mark it with a cross

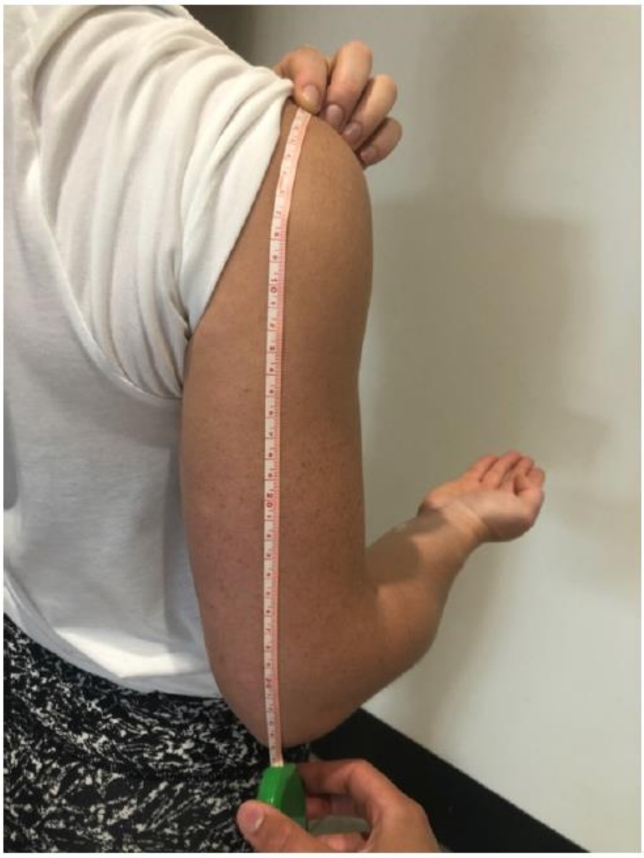

★ Here, the distance between the tip of the shoulder and the elbow is 35cm

4. Put the tape measure on the mark made at the shoulder and drop it down to the mark made at the tip of the elbow

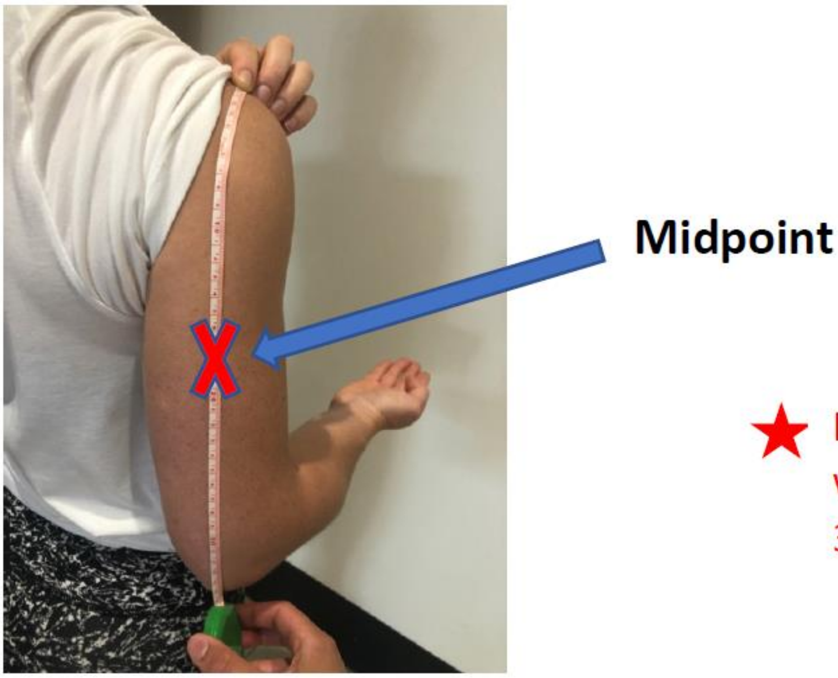

★ In this case, the midpoint would be 17.5cm, since 35cm / 2 = 17.5cm

5. Mark the **midpoint** between the two landmarks (**midpoint** = the middle point between the tip of the shoulder and the tip of the elbow)

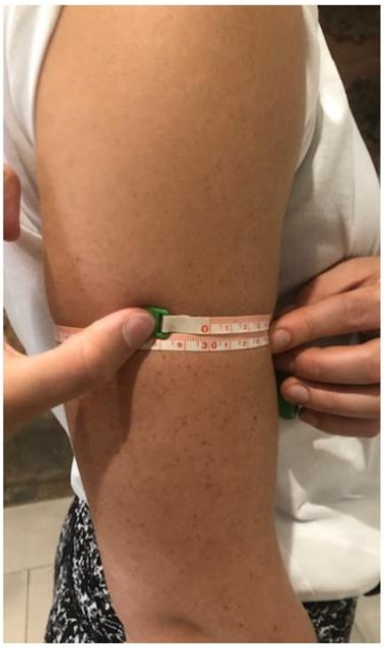

★ The tape should be right against the skin, not too loose and not too tight

★ The number that matches up with the **zero** is the arm circumference (in this case, 30cm)

Part 2: Measuring Circumference

1. Relax your elbow so that your arm is dangling freely to the side
2. Use the tape measure (make sure tape measure is **parallel to the floor**) to measure circumference at the marked mid-point (from step 5 above).
3. Record the arm circumference to the nearest 0.1cm
